# Interleukin-1 receptor 1 knockout has no effect on amyloid deposition in Tg2576 mice and does not alter efficacy following Aβ immunotherapy

**DOI:** 10.1186/1742-2094-3-17

**Published:** 2006-07-26

**Authors:** Pritam Das, Lisa A Smithson, Robert W Price, Vallie M Holloway, Yona Levites, Paramita Chakrabarty, Todd E Golde

**Affiliations:** 1 Department of Neurosciences, Mayo Clinic College of Medicine, 4500 San Pablo Road, Jacksonville, FL 32224, USA

## Abstract

**Background:**

Microglial activation has been proposed to facilitate clearance of amyloid β protein (Aβ) from the brain following Aβ immunotherapy in amyloid precursor protein (APP) transgenic mice. Interleukin-1 receptor 1 knockout (IL-1 R1-/-) mice are reported to exhibit blunted inflammatory responses to injury. To further define the role of IL-1-mediated inflammatory responses and microglial activation in this paradigm, we examined the efficacy of passive Aβ immunotherapy in Tg2576 mice crossed into the IL-1 R1-/- background. In addition, we examined if loss of IL-1 R1-/- modifies Aβ deposition in the absence of additional manipulations.

**Methods:**

We passively immunized Tg2576 mice crossed into the IL-1 R1-/- background (APP/IL-1 R1-/- mice) with an anti-Aβ1-16 mAb (mAb9, IgG2a) that we previously showed could attenuate Aβ deposition in Tg2576 mice. We also examined whether the IL-1 R1 knockout background modifies Aβ deposition in untreated mice. Biochemical and immunohistochemical Aβ loads and microglial activation was assessed.

**Results:**

Passive immunization with anti-Aβ mAb was effective in reducing plaque load in APP/IL-1 R1-/- mice when the immunization was started prior to significant plaque deposition. Similar to previous studies, immunization was not effective in older APP/IL-1 R1-/- mice or IL-1 R1 sufficient wild type Tg2576 mice. Our analysis of Aβ deposition in the untreated APP/IL-1 R1-/- mice did not show differences on biochemical Aβ loads during normal aging of these mice compared to IL-1 R1 sufficient wild type Tg2576 mice.

**Conclusion:**

We find no evidence that the lack of the IL-1 R1 receptor influences either Aβ deposition or the efficacy of passive immunotherapy. Such results are consistent with other studies in Tg2576 mice that suggest microglial activation may not be required for efficacy in passive immunization approaches.

## Background

Direct immunization with aggregated amyloid β protein (Aβ) and passive immunization with anti-Aβ antibodies (Abs) reduce plaque burden in Alzheimer's disease (AD) mouse models and improve cognitive deficits present in those models [1-5]. Although no adverse effects of immunization were noted in earlier studies, more recent data in mice indicate that there is the potential of exacerbation of cerebral-amyloid angiopathy (CAA) associated microhemmorhages in certain mouse strains following passive immunization with certain anti-Aβ antibodies [6-8]. An active immunization trial in humans was initiated using fibrillar Aβ42+QS-21 adjuvant (AN-1792) but was halted due to a meningio-encephalitic presentation in ~6% of individuals [9-11]. Reports of individuals enrolled in the trial suggest that those subjects who developed modest anti-plaque antibody (Ab) titers did show some clinical benefit relative to subjects that did not develop detectable titers [9,11,12]. A small phase II study of AD patients administered human IVIG containing anti-Aβ Abs showed slight improvement in ADAScog following administration; however the clinical effect was modest and only a few subjects were evaluated [13].

Given the pre-clinical data, hints of efficacy in humans, and the lack of disease-modifying therapies for AD, Aβ immunotherapy or derivative approaches are still worthy of pursuing. However, the mechanism or mechanisms through which Aβ immunotherapy works remain enigmatic [14,15]. The amount of Aβ deposited when immunization is initiated, the AD mouse model used, and the properties of the anti-Aβ antibodies used, all affect the outcome [1,2,16-18]. One of the debates with respect to mechanism centers on peripheral versus a central action of the antibody [3,19,20]. There is evidence to support both mechanisms, and it will be a very difficult issue to definitively address this through additional experimentation. Another debate is in regard to the role of microglia activation. Several groups report transient or stable enhancements of microglia activation associated with Aβ removal; others do not [1,21-23]. In postmortem human tissue from AD patients who had received the AN-1792 vaccine, Aβ-laden microglia were noted in areas where Aβ clearance is hypothesized to have occurred [24]. Thus, microglial activation has been proposed to facilitate removal of Aβ from the brain following vaccination.

The IL-1 superfamily (including IL-1β, IL-1α and IL-18) is a group of cytokines that exhibit a large number of biological responses [25]. Interleukin-1β is a key mediator of host response to infections and a primary cause of inflammation [25]. In vivo, IL-1β is elevated during infections and in several chronic inflammatory diseases such as arthritis, scleroderma, systemic lupus erythematosus, vasculitis, sepsis, septic shock, and atherosclerotic lesions as well as in brains of AD patients [25]. As least two IL-1 receptors (IL-1R) have been identified: type I and type II receptors (IL-RI and IL-RII) [26]. IL-1β binds IL-1RI and upon IL-1 binding, IL-1RI recruits the accessory protein IL-1R-AcP, and initiates a stimulatory signal transduction cascade [26]. IL-1RII acts as a decoy receptor and competes with IL-1RI to down-modulate IL-1 activity [27]. In AD and Down's syndrome, IL-1β production is increased in microglial cells in the vicinity of amyloid plaques [28,29]. Initial studies examining the association of polymorphisms in the IL-1 and IL-1 receptor genes showed positive association of certain alleles with AD risk [30-34]. However, like many AD genetic association studies, subsequent studies failed to confirm the initial association. Meta-analyses of all studies on IL-1α and β linkage show no evidence for association of these loci with AD . A recent report shows that activation of microglia with secreted APP (sAPPα) results in a dose-dependent increase in secreted IL-1β [35]. Similarly, cortical neurons treated with IL-1β showed a dose-dependent increase in sAPPα secretion, elevated levels of α-synuclein and phosphorylated tau [35]. In APP transgenic mice, IL-1 reactivity and other inflammatory markers are increased in microglial cells surrounding amyloid deposits during various stages of amyloid deposition in these mice [36,37]. Another member of the IL-1 superfamily, IL-1 receptor antagonist (IL-1Ra) [38], is also synthesized and released in parallel to IL-1β, IL-1α, and IL-18. IL-1Ra binds to IL-1RI and blocks IL-1 dependent signal transduction, thus functioning as an endogenous, IL-1 selective inhibitor of inflammation [38]. Interestingly, IL-1Ra knockout mice show enhanced microglial activation and neuronal damage following intracerebroventricular infusion of human Aβ [39]. Collectively, these data suggest that IL-1 is a key mediator of microgliosis and subsequent inflammatory responses following Aβ deposition as well as in the production of substrates necessary for neuropathological changes seen in AD.

To gain additional insight into the role of IL-1 signaling on microglial activation, on IL-1-mediated inflammatory responses following Aβ vaccination, and on Aβ deposition during normal aging, we used interleukin-1 receptor 1-knockout (IL-1 R1-/-) mice [40-42] that were crossed to APP Tg2576 transgenic mice (APP/IL-1 R1-/-). The IL-1 R1-/- mice lack the type 1 interleukin-1 receptor, but develop normally. Moreover, with a few exceptions, these mice are normal, showing alterations in IL-1-mediated immune response to certain stimuli. Following penetrating brain injury in IL1-R1-/- mice, fewer amoeboid microglia/macrophages are present near the sites of injury, astrogliosis is mildly abrogated and cyclooxygenase-2 (Cox-2) and IL-6 expression are reduced [42]. In another report, IL-1 R1-/- mice failed to respond to IL-1 in several assays, including IL-1-induced IL-6 and E-selectin expression, and IL-1-induced fever and acute-phase responses to turpentine [41]. These data in IL-1 R1-/- mice demonstrate that IL-1 R1 is critical for most IL-1-mediated signaling events tested. We performed passive immunization with an anti-Aβ mAb in Tg2576 mice crossed into the IL-1 R1-/- background (APP/IL-1 R1-/-), and determined whether microglial activation and consequent inflammatory responses are necessary for Aβ reduction. These studies show that passive immunization with anti-Aβ mAb is effective in reducing plaque load in APP/IL-1 R1-/- mice when the immunization is started prior to significant plaque deposition and thus support our general hypothesis that microglial activation may not be required for efficacy of immunization in Tg2576 mice.

## Methods

### Mice breeding strategy

Tg2576 [43] were bred into the IL-1 R1-knockout background (B6.129S7-Il1r1tm1Imx, Jackson Laboratories) as follows; male Tg2576 (C57BL/6.SJL) were initially crossed with IL-1 R1-/- females (B6.129S7). We then backcrossed the F1 Tg2576 × IL-1R1+/- males with female IL-1 R1-/-. These crosses generated the F2 Tg2576 × IL-1R1-/- mice (APP/IL-1 R1-/-) and Tg2576 × IL-1R1+/- littermates (APP/IL-1 R1+/-), which were used in all experiments. All animal experimental procedures were performed according to Mayo Clinic Institutional Animal Care and Use Committee guidelines. All animals were housed three to five to a cage and maintained on *ad libitum *and water with a 12 h light/dark cycle.

### Passive immunizations

Groups of APP/IL-1 R1-/- mice and APP/IL-1 R1+/- littermates (males and females, 6-month-old or 12-month-old, n = 3-5/group) were immunized intraperitoneally (i.p.) with 500 μg of mAb9 (Aβ1-16 specific, IgG2a) in saline once every 2 weeks for 3 months. Control mice received 500 μg of purified mouse IgG in saline.

### ELISA analysis of extracted Aβ

At sacrifice, the brains of mice were divided by midsagittal dissection, and 1 hemibrain was used for biochemical analysis as described previously [18]. Briefly, each hemibrain (150 mg/ml wet wt) was extracted in 2% SDS with protease inhibitors using a polytron and centrifuged at 100,000 *g *for 1 hour at 4°C. Following centrifugation, the supernatant was collected, which represented the SDS-soluble fraction. The resultant pellet was then extracted in 70% FA, using a probe sonicator, centrifuged at 100,000 *g *for 1 hour at 4°C, and the supernatant collected (the FA fraction). Extracted Aβ was then measured using a sandwich ELISA system as described before [18]; Aβ 42-capture with mAb 2.1.3 (mAb40.2,) and detection with HRP-conjugated mAb Ab9 (human Aβ1-16 specific); Aβ40- capture with mAb Ab9 and detection with HRP-conjugated mAb 13.1.1 (mAβ40.1)

### Immunohistology

Hemibrains of mice were fixed in 4% paraformaldehyde in 0.1 M PBS (pH 7.6) and then stained for Aβ plaques as described previously [18]. Paraffin sections (5 μm) were pretreated with 80% FA for 5 minutes, boiled in water using a rice steam cooker, washed, and immersed in 0.3% H2O2 for 30 minutes to block intrinsic peroxidase activity. They were then incubated with 2% normal goat serum in PBS for 1 hour, with 33.1.1 (Aβ1-16 mAb) at 1 μg/ml dilution overnight, and then with HRP-conjugated goat anti-mouse secondary mAb (1:500 dilution; Amersham Biosciences) for 1 hour. Sections were washed in PBS, and immunoreactivity was visualized by 3,3'-diaminobenzidine tetrahydrochloride (DAB) according to the manufacturer's specifications (ABC system; Vector Laboratories). Adjacent sections were stained with 4% thioflavin-S for 10 minutes. Free-floating 4% paraformaldehyde-fixed, frozen tissue sections (30 μM) were stained for the presence of activated microglia with rat anti-mouse CD45 (1:3000; Serotec, Oxford, UK), followed by detection with anti-rat-HRP (ABC system, Vector Labs), and then counterstained with Thio-S as described previously [23]. Four percent paraformaldehyde-fixed, paraffin-embedded sections were stained for activated microglia using anti-Iba1 (1:3000; Wako Chemicals) and for activated astrocytes using anti-GFAP (1:1000, Chemicon).

### Quantitation of amyloid plaque burden

Computer-assisted quantification of Aβ plaques was performed using he MetaMorph 6.1 software (Universal Imaging Corp, Downington, PA). Serial coronal sections stained as above were captured, and the threshold for plaque staining was determined and kept constant throughout the analysis. For analysis of plaque burdens in the passive immunization experiments, immunostained plaques were quantified (proportional area of plaque burden) in the neocortex of the same plane of section for each mouse (~10 sections per mouse). All of the above analyses were performed in a blinded fashion.

### Statistical analysis

One-way ANOVA followed by Dunnett's multiple comparison tests were performed using the scientific statistic software Prism (version 4; GraphPad).

## Results

### Interleukin-1 receptor 1 knockout has no effect on Aβ loads in Tg2576 mice

To investigate whether the lack of IL-1 R1 had any effect on Aβ deposition, we analyzed biochemically extractable Aβ levels and immuno-reactive plaque burdens in Tg2576 mice crossed to IL-1 R1-/- mice (APP/IL-1 R1-/-). APP/IL-1 R1-/- mice were compared to APP/IL-1 R1+/- hemizygous littermates to control for differences in the background genes, as a result of our breeding strategy (Tg2576 in F1 C57BL/6.SJL background and IL1-R1-/- mice in B6.129S7 background). Thus, APP/IL-1 R1-/- mice and APP/IL-1 R1+/- hemizygous littermates generated are in similar mixed C57BL/6.SJL and C57BL/6.129S7 backgrounds. We have also compared the crossed mice to wild type Tg2576 mice (referred to as IL-1 R1+/+) in various measurements, though these mice are in a different background (F2 C57BL/6.SJL).

**Figure 1:**
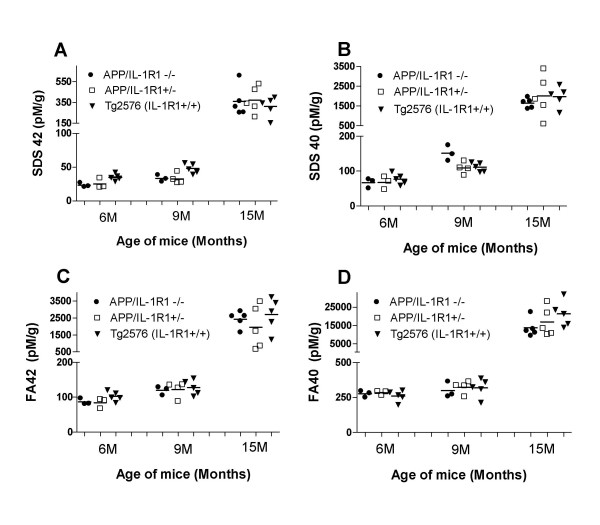
Aβ levels in APP/IL-1 R1-/- mice, APP/IL-1 R1+/-littermates and wild type Tg2576 mice at 6 months, 9 months and 15 months of age. Groups of mice were killed at the indicated time points and both SDS-soluble (SDS) and SDS-insoluble, formic acid extractable (FA) fractions of Aβ40 and Aβ42 were measured by capture ELISA.

**Figure 2:**
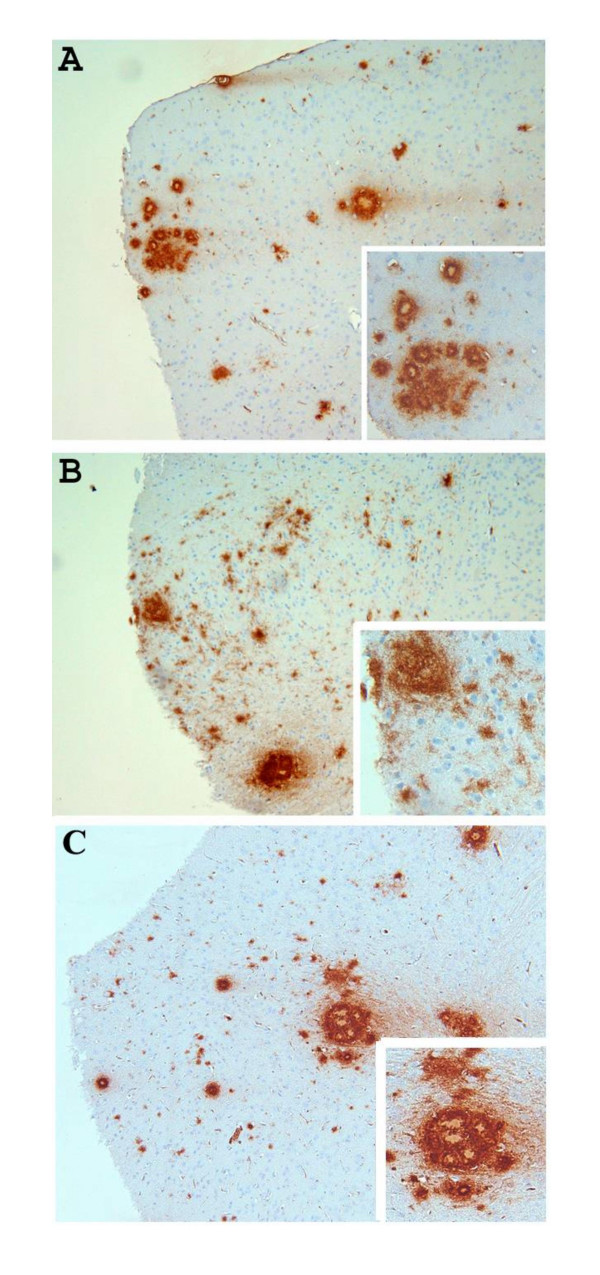
Representative pictures of immunostained Aβ plaques (stained with anti-Aβ antibody) in the neocortex of **(A) **a 15-month-old APP/IL-1 R1+/- mouse; **(B) **a 15-month-old APP/IL-1 R1-/- mouse; and **(C) **a 15-month-old wild type Tg2576 mice (IL_1 R1+/+). (A, B, C, magnification = 100×, insert shows enlargement of Aβ plaques).

Groups of mice at various ages (6 months, 9 months and 15 months of age) were killed and the levels of both SDS-soluble (SDS) and SDS-insoluble FA-extractable fractions of Aβ40 and Aβ42 were analyzed by ELISA. As shown in Figure 1, there were no significant differences in the amounts of extractable Aβ in all three ages groups tested when we compared Aβ levels in APP/IL-1 R1-/-, APP/IL-1 R1+/- littermates and wild type Tg2576 mice: SDS Aβ42 (Figure 1A), SDS Aβ40 (Figure 1B), FA Aβ42 (Figure 1C), and FA Aβ40 (Figure 1D). To further examine whether there were alterations in deposited Aβ plaques in these mice, coronal sections of each mouse hemibrain were analyzed for changes in immunostained Aβ plaque loads. Quantitative image analysis of amyloid plaque burden in all age groups revealed no significant differences (data not shown). However, in 2 of 7 mice analyzed in the 15-month-old APP/IL-1 R1-/-, there was atypical Aβ plaque staining. An appreciable increase in diffuse immuno-reactive Aβ plaques (Figure 2B) in the neocortex of these 2 mice was noted when compared to the 15-month-old APP/IL-1 R1+/- littermates (Figure 2A) or wild type Tg2576 mice (Figure 2C), which deposit more dense-cored Aβ plaques at this age.

### Passive immunotherapy is effective in young APP/IL-1 R1-/- mice

**Figure 3:**
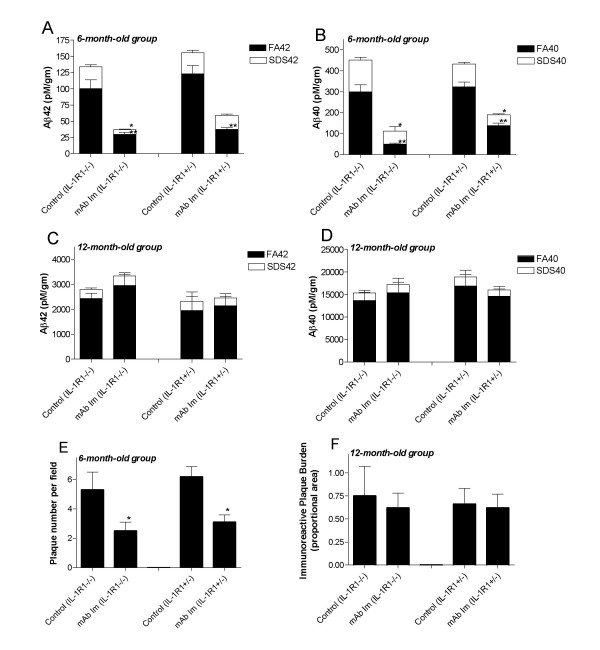
**A and B**. Aβ levels were significantly reduced following mAb9 immunizations initiated in 6-month-old APP/IL-1 R1-/- mice as well as APP/IL-1 R1+/- mice (n = 3/group). **C and D**. Aβ levels were not significantly altered following mAb9 immunizations initiated in 12-month-old APP/IL-1 R1-/- mice and APP/IL-1 R1+/- mice (n = 3–5/group). Mice were killed after immunization with 500 μg of mAb9 every other week for 3 months, and both SDS-soluble (SDS) and SDS-insoluble, formic acid extractable (FA) fractions of Aβ40 and Aβ42 were measured by capture ELISA. **E and F**. Quantitative image analysis of amyloid plaque burden in the neocortex of mAb9 immunizations initiated in 6-month-old APP/IL-1 R1-/- mice **(E) **and mAb9 immunizations initiated in 12-month-old APP/IL-1 R1-/- mice **(F)**. (*, ** P < 0.05 t-test)

To examine the effects of microglial activation on Aβ immunotherapy, we examined the effects of passive immunization with an anti-Aβ monoclonal antibody (mAb9) in APP/IL-1 R1-/-mice. Two experimental paradigms were used: i) a prevention study, in which passive immunization was performed in 6-month-old mice, which at this time have minimal Aβ deposition, and ii) a therapeutic study, in which immunotherapy was performed using 12-month-old mice, which have moderate levels of preexisting Aβ deposits. Both groups of mice were treated for 3 months then killed; and biochemical and immunohistochemical methods were used to analyze the effect of immunotherapy. Following passive immunization with mAb9 initiated in the 6-month-old mice (prevention study), Aβ levels were significantly attenuated in both the APP/IL-1 R1-/- and APP/IL-1 R1+/- littermates (Figure 3). Both the SDS-extractable Aβ levels (>50% reduction in SDS Aβ; Figure 3A and 3B) and formic acid- (FA-) solubilized, SDS-insoluble material (>50% reduction in FA Aβ; Figure 3A and 3B) were reduced in these mice. Quantitative image analysis of immunostained sections also showed a significant decrease in Aβ deposition in both groups (as measured by plaque numbers per field, Figure 3E). In contrast, passive immunization with mAb9, initiated in the 12-month-old mice (therapeutic study) had no significant effect on biochemically extracted Aβ levels (Figure 3C and 3D) or immuno-reactive Aβ; plaque loads (Figure 3F), in the both the APP/IL-1 R1-/- or APP/IL-1 R1+/- littermates.

### Interleukin-1 receptor 1 knockout has no effect on microglial reactivity surrounding Aβ plaques

**Figure 4:**
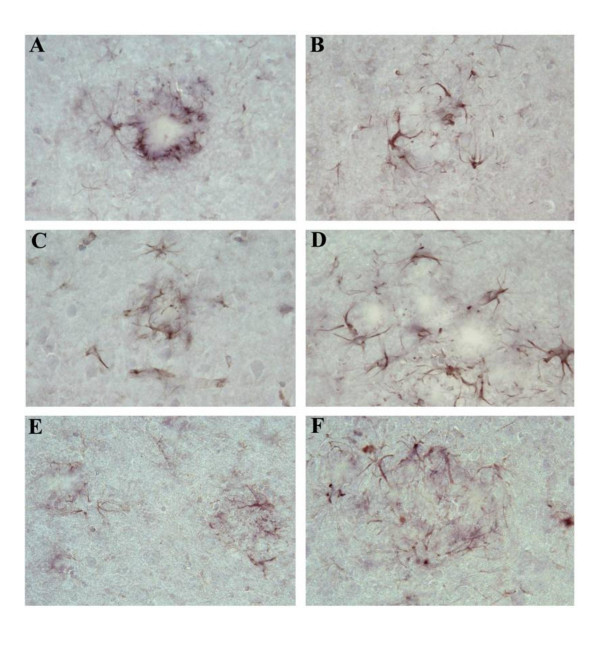
Representative pictures of Thioflavin-S-stained Aβ plaques (lightly stained areas) decorated with ramified microglia immunostained with anti-mouse CD45 (black stain) in the neo cortex of untreated **(A) **9-month-old APP/IL-1 R1-/- and **(B) **15-month-old APP/IL-1 R1-/-; **(C) **9-month-old APP/IL-1 R1+/- and **(D) **15-month-old APP/IL-1 R1+/-; **(E) **9-month-old wild type Tg2576 mice (IL-1 R1+/+) and **(F) **15-month-old wild type Tg2576 mice (IL-1 R1+/+). (A, B, C, D, E, F magnification = 400×).

**Figure 5:**
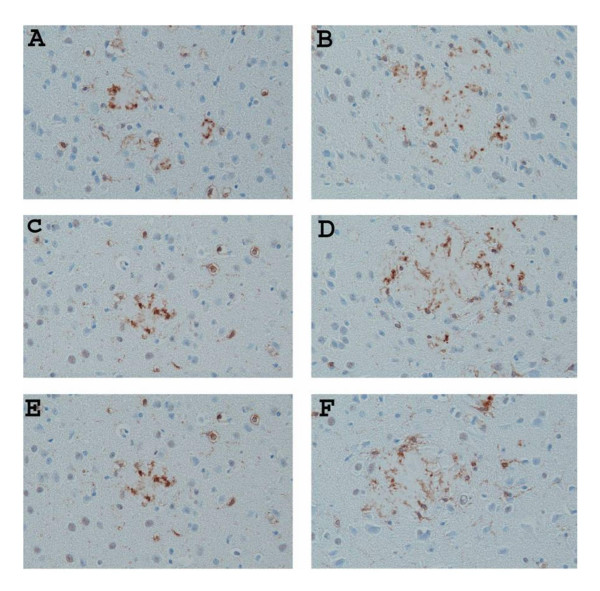
Representative pictures of Thioflavin-S-stained Aβ plaques (lightly stained areas) decorated with microglia immunostained with anti-Iba1 (brown stain) in the neocortex of untreated **(A) **9-month-old APP/IL-1 R1-/- and **(B) **15-month-old APP/IL-1 R1-/-; **(C) **9-month-old APP/IL-1 R1+/- and **(D) **15-month-old APP/IL-1 R1+/-; **(E) **9-month-old wild type Tg2576 mice (IL-1 R1+/+) and **(F) **15-month-old wild type Tg2576 mice (IL-1 R1+/+). (A, B, C, D, E, F magnification = 400×).

**Figure 6:**
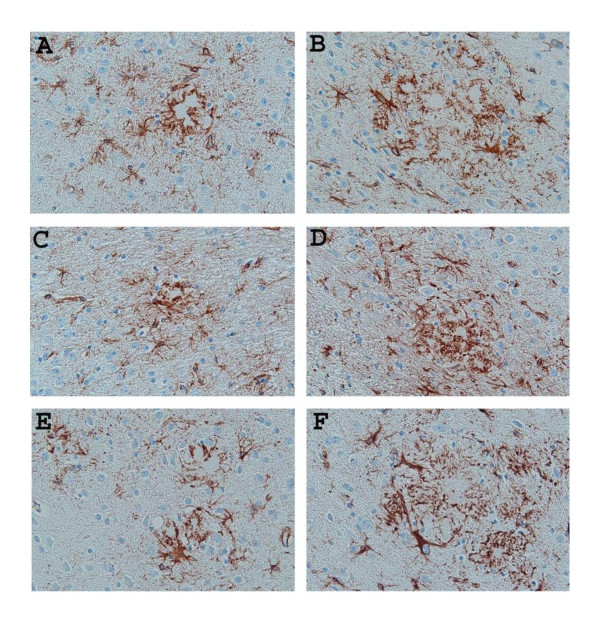
Representative pictures of Thioflavin-S-stained Aβ plaques (lightly stained areas) decorated with activated astrocytes immunostained with anti-GFAP (brown stain) in the neocortex of untreated **(A) **9-month-old APP/IL-1 R1-/- and **(B) **15-month-old APP/IL-1 R1-/-; **(C) **9-month-old APP/IL-1 R1+/- and **(D) **15-month-old APP/IL-1 R1+/-; **(E) **9-month-old wild type Tg2576 mice (IL-1 R1+/+) and **(F) **15-month-old wild type Tg2576 mice (IL-1 R1+/+). (A, B, C, D, E, F magnification = 400×).

To access whether the IL-1 R1-/- phenotype affected the state of microglial activation, and astrocyte reactivity, particularly, glial reactivity surrounding amyloid plaques, we compared the intensity of staining of microglia using antibodies against CD45, a marker for activated microglia that has been shown to be present on activated microglia surrounding amyloid plaques in APP transgenic mice [44] and Iba1, the ionized calcium-binding adaptor molecule 1, which is expressed selectively in activated microglia/macrophages [45]. For CD45 staining, coronal sections from both unmanipulated APP/IL-1 R1-/-, APP/IL-1 R1+/- littermates and wild type Tg2576 mice (IL-1 R1 +/+) at 9-months and 15-months of age were used for staining. As shown in Figure 4, there were abundant numbers of CD45 immuno-reactive microglia present, surrounding Aβ plaques from the 9-month-old APP/IL-1 R1-/- (Figure 4A), APP/IL-1 R1+/- littermates (Figure 4C) and wild type Tg2576 mice (Figure 4E) with no obvious differences in the CD45 reactivity in these activated microglial cells. Greater numbers of immuno-reactive microglia were present surrounding plaques in the 15-month-old mice, but again, there were no discernable differences in the density/CD45 reactivity in these microglial when we compared sections from the 15-month-old APP/IL-1 R1-/- (Figure 4B) vs. 15-month-old APP/IL-1 R1+/- littermates (Figure 4D) or wild type Tg2576 mice (Figure 4F). Similar results were seen when we compared the CD45 reactivity of microglia in mice that were passively immunized with mAb9 vs. controls, i.e., there were no differences in microglial reactivity using CD45 staining comparing immunized mice vs. controls in both groups (data not shown). For anti-Iba1 antibody staining, we compared coronal sections from unmanipulated APP/IL-1 R1-/-, APP/IL-1 R1+/- littermates and wild type Tg2576 mice (IL-1R1+/+) at 9 months and 15 months of age. As shown in Figure 5, anti-Iba1 staining was readily detected in microglia surrounding Aβ plaques in all three groups of mice tested comparing both 9-month-old and 15-month-old mice (Figure 5). Similar to CD45 staining, there were no discernable differences in the Iba1 reactivity in microglial cells comparing the APP/IL-1 R1-/-, APP/IL-1 R1+/- littermates and wild type Tg2576 mice (IL-1R1+/+) mice. For staining of activated astrocytes, we used an anti-GFAP antibody and compared immunoreactivity using coronal sections from unmanipulated APP/IL-1 R1-/-, APP/IL-1 R1+/- littermates and wild type Tg2576 mice (IL-1R1+/+) at 9 months and 15 months of age as before. As shown in Figure 6, there was robust anti-GFAP reactivity on activated astrocytes surrounding Aβ plaques in all three groups of mice tested (Figure 6). Again, similar to the microglial staining pattern, there were no discernable differences in the GFAP reactivity on astrocytes in all three groups of mice tested.

## Discussion

Despite multiple studies of anti-Aβ immunotherapy in mice, there is still no consensus on how anti-Aβ immunotherapy works [14,15], particularly as it relates to the role of microglial activation. It was originally proposed that Aβ immunization triggers phagocytosis of antibody-bound Aβ immune complexes via microglial FcR. After immunization, increased number of microglial cells stained with anti-Aβ antibodies were observed [1]. Indeed, using an *ex vivo *strategy, it was shown that anti-Aβ antibodies induce phagocytosis of Aβ plaques [2]. Importantly, Fab fragments of these antibodies fail to induce Aβ phagocytosis, suggesting that the enhanced uptake is attributable to FcR [2]. Subsequent studies have shown that at least in Tg2576 APP mice, a role for enhanced phagocytosis of mAb:Aβ complexes via the FcR can largely be ruled out, since Aβ1-42 immunization in Tg2576 × FcRγ-/- crossed mice was effective in reducing Aβ loads [23]. Additional studies now show that an intact mAb (and therefore FCR interactions) is not required for efficacy; since Fab fragments [46] and scFv fragments (Levites and Golde, unpublished observation) are efficacious in immunotherapy. Several groups have reported that following Aβ immunotherapy, there are transient or stable enhancements of microglial activation associated with Aβ removal; whereas others do not find this [1,21-23]. Furthermore, in humans receiving the AN-1792 vaccine, Aβ-laden microglia have been noted in postmortem studies [24]. Although antibody and microglial Fc receptor-mediated interactions have been suggested to activate microglia following vaccinations, other inflammatory consequences may play a role in this paradigm. Based on published reports, it has been suggested that clearance of amyloid deposits in patients enrolled in the AN-1792 trial may have been due to an adverse inflammatory response to the vaccine rather than due to the anti-Aβ antibodies [47]. This proposition may be supported by some recent reports, wherein induction of experimental autoimmune encephalitis (EAE) and nasal vaccination with glatiramer acetate reportedly decrease amyloid plaques in APP transgenic mice [48]. Another report by the same group shows that, in mice over expressing IFN-gamma in the CNS, amyloid vaccination lead to meningoencephalitis and T cell-dependent clearance of amyloid plaques from the brain [49]. Both of these reports provide evidence that peripheral inflammatory responses and CNS autoreactive T cells may play a role in vaccination-induced clearance of plaques. Furthermore, some recent reports have indicated that inflammatory insults, either by injecting LPS directly into the brain [44,50] or overexpression of TGF-β in the CNS [51], can result in reductions of amyloid deposits. Enhanced microglial activation was noted in both of these reports and is suggested to contribute to the clearance of amyloid deposits.

In this report, we sought to determine the role of IL-1-mediated microglial activation on IL-1-mediated inflammatory responses following Aβ vaccination and on Aβ deposition during normal aging using interleukin-1 receptor 1-knockout (IL-1 R1-/-) mice [40-42] that were crossed to APP Tg2576 transgenic mice (APP/IL-1 R1-/-). We first tested the efficacy of Aβ immunization in APP/IL-1 R1-/- mice. Our results show that passive immunization with an anti-Aβ mAb is effective in reducing plaque loads both in APP/IL-1 R1-/- mice and APP/IL-1 R1+/- littermates, when immunization is started prior to significant plaque deposition. However, as we have seen previously, immunization was not efficacious in mice that have pre-existing Aβ loads [17,18,52]. Thus, these results support our general hypothesis that microglial activation may not be required for efficacy of immunization in Tg2576 mice. The lack of IL-1 R1 (in -/- mice) did not significantly alter Aβ deposition in untreated mice. There were no significant differences in total extractable Aβ levels or overall histochemical loads, at any time, between the APP/IL-1 R1-/- mice and APP/IL-1 R1+/- littermates compared to wild type Tg2576 mice (IL-1 R1+/+). Curiously, in 2 of 7 15-month-old APP/IL-1 R1-/- mice examined, an unusual pattern of Aβ plaque staining was noted, with an abundance of diffuse immuno-reactive Aβ plaques in the neocortex of these mice. It is not clear at this time whether this unusual pattern of diffuse Aβ deposits is due to the IL-1 R1-/- phenotype or some mouse background effect. We then examined the effects of IL-1 R1 knockout on the state of microglial activation and astrogliosis surrounding amyloid plaques deposits. For microglial staining, we used two well characterized markers for microglial activation, anti-mouse CD45 and Iba1, and for activated astrocytes we used anti-GFAP staining. Our results show that there were abundant numbers of CD45 and Iba1 immuno-reactive microglia present, surrounding Aβ plaques in APP/IL-1 R1-/-, APP/IL-1 R1+/- and wild type Tg2576 mice (IL-1 R1+/+), with no significant differences in the immuno-reactivity of staining using these markers. Similarly, robust GFAP staining was seen in all three groups of mice analyzed, with no significant differences seen in the GFAP immuno-reactivity comparing all three groups of mice.

Based on our immuno-staining analysis, we were not able to ascertain whether abrogated IL-1 signaling in the IL-1 R1-/- mice blunted the inflammatory microglial response or astrogliosis in the region of deposited Aβ plaques. Previous experiments in IL-1 R1-/- mice have shown abrogated IL-1-mediated responses following acute inflammatory stimuli. In a stab wound model of injury in the brain, IL1-R1-/- mice had fewer amoeboid microglia/macrophages near the sites of injury, mildly abrogated astrogliosis and reduced expression of cytokines induced by IL-1 expression [42]. In another report, IL-1 R1-/- mice had reduced IL-6 and E-selectin expression, and reduced IL-1-induced fever and acute phase responses to turpentine [41]. However, IL-1 R1-/- mice do not differ from control mice in their responses to either a lethal challenge with D-galactosamine plus LPS or high dose LPS [40], indicating that IL-1 R1 signaling functions may not be necessary for the response to LPS. Thus, it is possible that the chronic nature of the microglial response during the course of amyloid deposition may abrogate any acute or subtle signaling events mediated through the IL-1 R1 receptor. Certainly, it is possible that other receptors for IL-1 may compensate for the lack for IL-1 R1 in this situation. Besides the IL-1 R1 and IL-1 RII receptors, the recently reported P2X7 receptor has also been implicated to be a key player in IL-1 signaling [53] and could compensate for the lack of IL-1R1 in IL-1 mediated signaling events. Alternatively, the microglial response to deposited Aβ may not require signaling through the IL-1 R1 receptor. The LPS receptor (CD14) [54,55], the scavenger receptor complex (CD36) [56] and toll-like receptors (TLR-2, TLR-4) [57] can directly activate microglia in response to amyloid deposition, possibly circumventing any IL-1 R1-mediated signaling events in the IL-1 R1-/- mice.

Like our previous studies, these studies suggest that microglial activation is not required for immunization to work in Tg2576 mice, although this should not be viewed as definitive. As indicated above, in the IL-1 R1-/- mice, microgliosis and astrogliosis are mildly abrogated at best and do not result in microglial paralysis. Thus experiments using recently developed CD11b-HSVTK mice. developed by Aguzzi and colleagues [58]. that enable selective killing of microglia cells may provide more definitive results.

## Competing interests

The author(s) declare that they have no competing interests.

## Authors' contributions

PD conceived the design of the study, performed experimental analysis and data interpretation and prepared the manuscript. LAS bred and maintained the IL-1 R1-/- mice, performed immunizations, harvested tissues, performed CD45 staining. RWP performed the Aβ ELISA. VMH performed the image quantification and immunohistology. YL performed Aβ plasma ELISA and aided in the preparation of the manuscript. PC performed Iba1 immunostaining and APP western blotting. TEG conceived the design of the study, aided in the preparation of the manuscript, and provided critical analysis of the manuscript.
